# Biosynthesis and Biological Activities of Newly Discovered Amaryllidaceae Alkaloids

**DOI:** 10.3390/molecules25214901

**Published:** 2020-10-23

**Authors:** Seydou Ka, Manoj Koirala, Natacha Mérindol, Isabel Desgagné-Penix

**Affiliations:** 1 Department of Chemistry, Biochemistry and Physics, Université du Québec à Trois-Rivières, 3351, boul. des Forges, C.P. 500, Trois-Rivières, QC G9A 5H7, Canada; Seydou.Ka@uqtr.ca (S.K.); Manoj.Koirala@uqtr.ca (M.K.); Natacha.Merindol@uqtr.ca (N.M.); 2 Groupe de Recherche en Biologie Végétale, Université du Québec à Trois-Rivières, 3351, boul. des Forges, C.P. 500, Trois-Rivières, QC G9A 5H7, Canada

**Keywords:** Amaryllidaceae alkaloids, specialized metabolism, biosynthesis, antitumor, anti-cholinesterase, antiviral, antiparasitic

## Abstract

Alkaloids are an important group of specialized nitrogen metabolites with a wide range of biochemical and pharmacological effects. Since the first publication on lycorine in 1877, more than 650 alkaloids have been extracted from Amaryllidaceae bulbous plants and clustered together as the Amaryllidaceae alkaloids (AAs) family. AAs are specifically remarkable for their diverse pharmaceutical properties, as exemplified by the success of galantamine used to treat the symptoms of Alzheimer’s disease. This review addresses the isolation, biological, and structure activity of AAs discovered from January 2015 to August 2020, supporting their therapeutic interest.

## 1. Introduction

The Amaryllidaceae species, belonging to the Asparagales monocot order, are a class of herbaceous, perennial, and bulbous flowering plants. The Amaryllidaceae plant family contains 85 genera and 1100 species that are widely distributed in the tropic and warm temperate regions of the globe [1]. In addition, the Amaryllidaceae plants are cultivated and exploited as ornamental plants for their beautiful flowers [2]. For centuries, Amaryllidaceae have been used in traditional medicine, such as the oil extracted from the daffodil *Narcissus poeticus*, used to treat uterine tumors [3]. Since the isolation of lycorine in 1877 (initially named narcissia) from *Narcissus pseudonarcissus* and in 1897 from *Lycoris radiata* [4,5,6], the structures of hundreds of Amaryllidaceae alkaloids (AAs) have been elucidated [1,3,7]. They are exploited for their wide range of biological potentials including antitumor, antiviral, antibacterial, antifungal, antimalarial, anti-acetylcholinesterase (anti-AChE), analgesic, and cytotoxic activities [8,9]. Currently, in terms of commercial success, galantamine, widely occurring in the Amaryllidaceae plants, has been approved as an AChE inhibitor by the United States Food and Drug Administration to treat the symptoms of Alzheimer’s disease (AD) [10]. Moreover, several other AAs, including lycorine, haemanthamine, and narciclasine have been used as lead molecules for anticancer research [11]. Thus, AAs represent an important resource for drug discovery.

This review addresses the isolation, biosynthesis, biological activities and structure activity of AAs discovered from January 2015 to August 2020.

## 2. Classification of Amaryllidaceae Alkaloids

**Figure 1:**
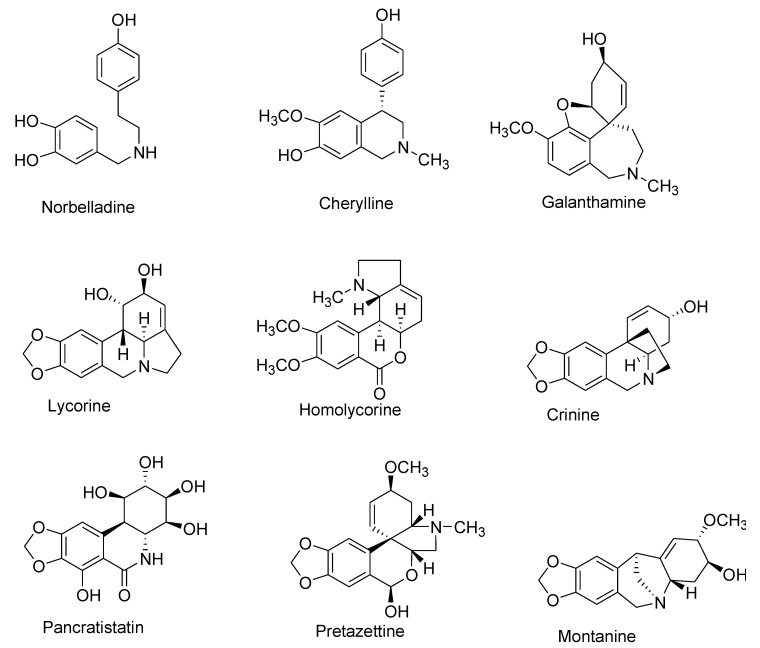
Representative Amaryllidaceae alkaloid structure for the main Amaryllidaceae alkaloid (AA)-types.

To date, more than 650 AAs have been reported, and their chemical library is still expanding [1,12,13,14,15,16,17,18,19,20,21,22,23,24]. Although diverse in structure, this plethora of AAs are categorized together as they share a common initial synthesis pathway. In previous literature, large numbers of AAs have been classified into different groups according to chemical characteristics, e.g., molecular skeleton and ring structure [1,3,8,25]. For this review, AAs were classified into 10 main groups instead, following a biochemical classification based on biogenetic lineage and ring type, to easily track the biosynthetic pathways [26] (Table 1, Figure 1). For example, haemanthamine and crinine were grouped together with respect to their biosynthetic origin and ring type even if they were previously categorized separately [11]. Some AAs with ring types different than those of group I to IX were classified in group X (or other-types) because they follow distinct biogenetic pathway, or because we cannot clearly indicate their biosynthetic origin (Table 1). Galanthindole contains a non-fused indole ring and might represent an artifact of homolycorine- or of pretazettine-type derivatives [27]. Ismine is considered to be a catabolic product from the haemanthamine-type skeleton, thus not a specific type of AA [28]. The latter examples demand further investigation on biogenetic origin and are not yet included on any particular type of AA. 

Some types of AA, such as plicamine and secoplicamine, are extracted in trace amounts exclusively from specific Amaryllidaceae species, such as Zephyranthes, but are classified in type X as they are rare, dinitrogenous members of AA, with a distinct biosynthetic linage [28,29,30,31]. Mesembrine alkaloids (also known as sceletium) have a distinct biosynthetic pathway, without norbelladine as key intermediate, they are usually extracted from Aizoaceae, but can be collected from several species of Amaryllidaceae [20]. Here, we concentrated exclusively on AAs that were discovered since 2015; hence, some alkaloids families are not represented.

## 3. Biosynthesis of Amaryllidaceae Alkaloids

Biosynthesis of AAs with their diverse and complex carbon skeleton involves a sequence of biochemical reactions such as oxidation, reduction, hydroxylation, methylation, phenol-phenol coupling, and oxide bridge formation. Although the complete AA biosynthetic pathway has not yet been elucidated, several steps with catalyzing enzymes can be predicted on the basis of the reaction types and enzyme families [2,26,32,33]. Here, we briefly discuss the AAs biosynthesis pathway and the enzymes involved.

**Figure 2:**
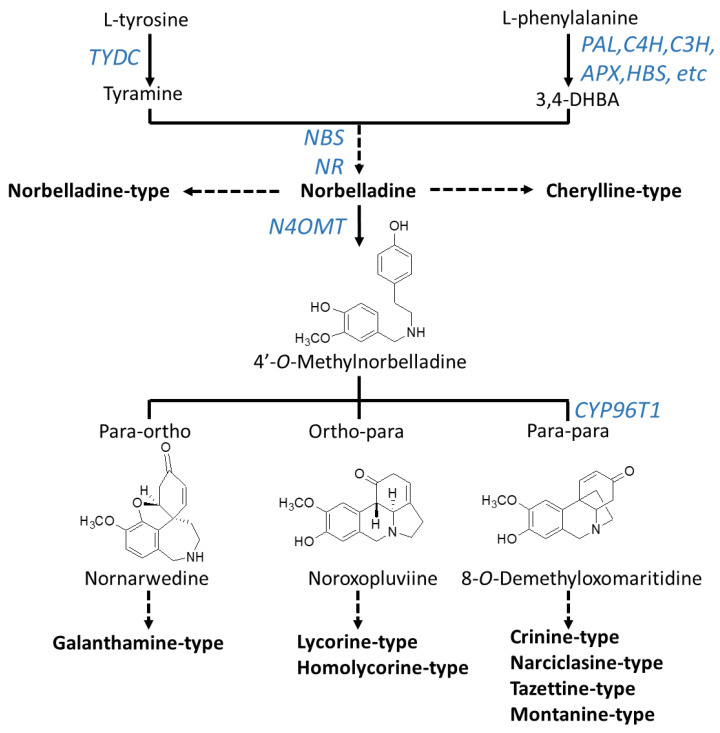
Biosynthesis pathway to major types of Amaryllidaceae alkaloids. Arrows without labeling reflect chemical reactions that have not been enzymatically characterized. Enzymes that have been identified are labeled in blue. A solid arrow symbolizes one enzymatic step whereas a broken arrow shows multiple enzymatic reactions. Chemical structures of precursors were added to clarify the regioselective phenol-phenol’ coupling reaction. Enzyme abbreviations: PAL, phenylalanine ammonia-lyase; C4H, cinnamate 4-hydroxylase; C3H, coumarate 3-hydroxylase; APX, ascorbate peroxidase; HBS, 4-hydroxybenzaldehyde synthase; TYDC, tyrosine decarboxylase; NBS, norbelladine synthase; NR, noroxomaritidine reductase; CYP96T1, cytochrome P450 monooxygenase 96T1.

Although novel AAs are still being discovered, radiolabeling experiments demonstrated that they all share a common biochemical pathway with a key intermediate; norbelladine, which is subsequently *O*-methylated, and then undergoes cyclization to give diverse basic skeletons of AAs (Figure 1; Figure 2) [34,35,36,37,38,39,40]. Norbelladine originates from the condensation of tyramine and 3,4-dihydroxybenzaldehyde (3,4-DHBA), molecules derived from the aromatic amino acids l-tyrosine and l-phenylalanine, respectively (Figure 2). The enzyme responsible for tyramine biosynthesis is the tyrosine decarboxylase (TYDC) (Figure 2). Two gene transcript variants of TYDC, named *TYDC1* and *TYDC2*, were identified from the transcriptome of different Amaryllidaceae species including *N*. *pseudonarcissus* [41], *Narcissus papyraceus* [42], *Lycoris radiata* [43], and *L. aureus* [44]. 

The pathway leading to 3,4-DHBA from l-phenylalanine involves a series of reactions known as the phenylpropanoid pathway which is phylogenetically spread in most plant species. In Amaryllidaceae, using precursor feeding experiments, it was reported that *trans*-cinnamic acid, *p*-coumaric acid, and caffeic acid were intermediate products that ultimately led to 3,4-DHBA [45,46]. Specifically, l-phenylalanine is converted to *trans*-cinnamic acid by the phenylalanine ammonia-lyase (PAL) (Figure 2). Several *PAL* gene transcripts were identified and characterized from different species of Amaryllidaceae [41,42,43,47,48,49]. Interestingly, two main phylogenetic *PAL* clusters were identified; the first one contained *PAL* transcripts ubiquitously expressed in Amaryllidaceae whereas the second cluster contained *PAL* transcripts with expression highest and correlating with organs where AAs accumulated [26]. This indicates that different *PAL* transcripts encode enzymes with distinct functions in the phenylpropanoid pathway and it suggests the role of the latter cluster in AA biosynthesis. Next, *trans*-cinnamic acid is hydroxylated to form *p*-coumaric acid by the cinnamate 4-hydroxylase (C4H), a cytochrome P450 of the CYP73 subfamily (cinnamate 4-hydroxylase, C4H) [50,51]. Transcripts encoding C4H were reported from several Amaryllidaceae species [41,42,43,49] including *C4H* from *L. radiate*, which was characterized as producing a region-specific 4-hydroxylation of *trans*-cinnamic acid [49]. The reactions catalyzed by PAL and C4H are also crucial steps in the biosynthesis of many phenylpropanoids such as flavonoids, linins, coumarins and stilbenes. From there, the enzymes and order of reactions leading to 3,4-DHBA are not known however it is hypothesized that it may involve the CYP98A3 named coumarate 3-hydroxylase (C3H) and/or the ascorbate peroxidase (APX) and/or the 4-hydroxybenzaldehyde synthase (HBS) [26] (Figure 2). A few studies have reported on the presence of phenolic acids such as caffeic, *p*-coumaric, and ferulic acids in *N. pseudonarcissus*, *N. poeticus* and *Galanthus nivalis* [52,53,54]. In addition, 3,4-DHBA was detected in plants outside the Amaryllidaceae family [55]. Collectively, this suggest that the initial reactions and enzymes of the phenylpropanoid pathway participate in the synthesis of the AA precursor 3,4-DHBA.

The condensation of tyramine and 3,4-DHBA leads to the formation of norbelladine catalyzed by the norbelladine synthase (NBS) and/or the noroxomaritidine reductase (NR) or a combination of these enzymes [56,57]. Norbelladine is the precursor to all AAs. For example, norbelladine can undergo different biochemical reactions such as methylation, hydroxylation, dehydration, cyclization and tautomerization to form cherylline-type AAs (Figure 2). Alternatively, norbelladine can be methylated by the norbelladine 4′-*O*-methyltransferase (N4OMT) to form 4′-*O*-methylnorbelladine [58]. 4′-*O*-methylnorbelladine cyclization in a regioselective phenol-phenol oxidative coupling forms the ortho-para’, para-para’ or para-ortho’ C-C coupled producing the various structural types of AAs (Figure 2). The para-ortho’ C-C coupling leads to galantamine-type AAs whereas the ortho-para’ phenol coupling elaborates lycorine- and homolycorine-types of AAs (Figure 2). The para-para phenol-phenol’ coupling reaction produces the crinine-, narciclasine-, tazettine- and montanine-types of AAs. A cytochrome P450 sequence was identified through comparative transcriptomics of *N.* sp. *aff. pseudonarcissus*, *Galanthus* sp., and *Galanthus elwesii*. Through heterologous expression and characterization, CYP96T1 formed the products (10b*R*,4a*S*)-noroxomaritidine and (10b*S*,4a*R*)-noroxomaritidine from 4′-*O*-methylnorbelladine supporting its involvement as a para-para’ C-C phenol coupling cytochrome P450 [59].

The core skeletons obtained from norbelladine, methylnorbelladine, and the phenol coupling steps form the basis of AA diversity. A complex network of enzyme catalyzing various types of reactions, such as C-C and C-O bond formations, *O*- and *N*-methylations, demethylations, hydroxylations, oxidations and reductions, yield the several hundred of structurally related AAs. Only a few of these enzymes are known to date and they are reported in Figure 2.

## 4. Occurrence of Amaryllidaceae Alkaloids

From January 2015 to August 2020, a total of 91 new AAs were isolated and identified from different plant species of the genus *Crinum*, *Zephyranthes*, *Narcissus*, *Galanthus*, *Hymenocallis*, *Nerine*, *Lycoris*, *Brunsvigia*, and *Hippeastrum* (Table 2).

**Figure 3:**
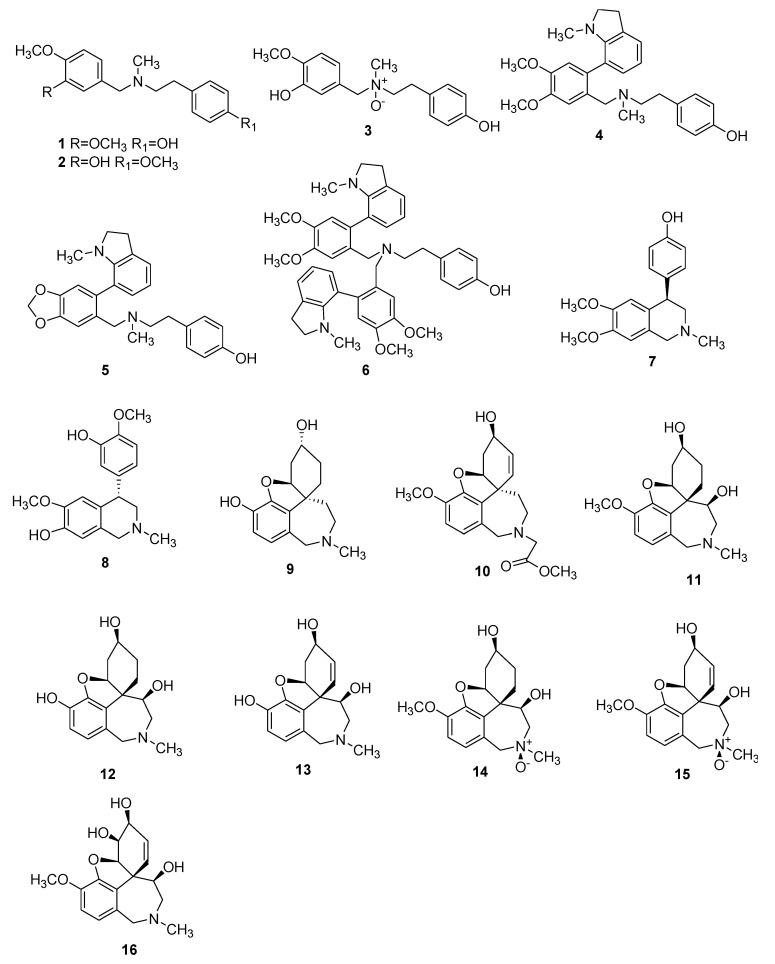
Chemical structure of novel Amaryllidaceae alkaloids of the norbelladine- (**1**–**6**), cherylline (**7**–**8**) and galantamine-type (**9**–**16**). Numbers in bold refer to the compounds depicted in Table 2.

**Figure 4:**
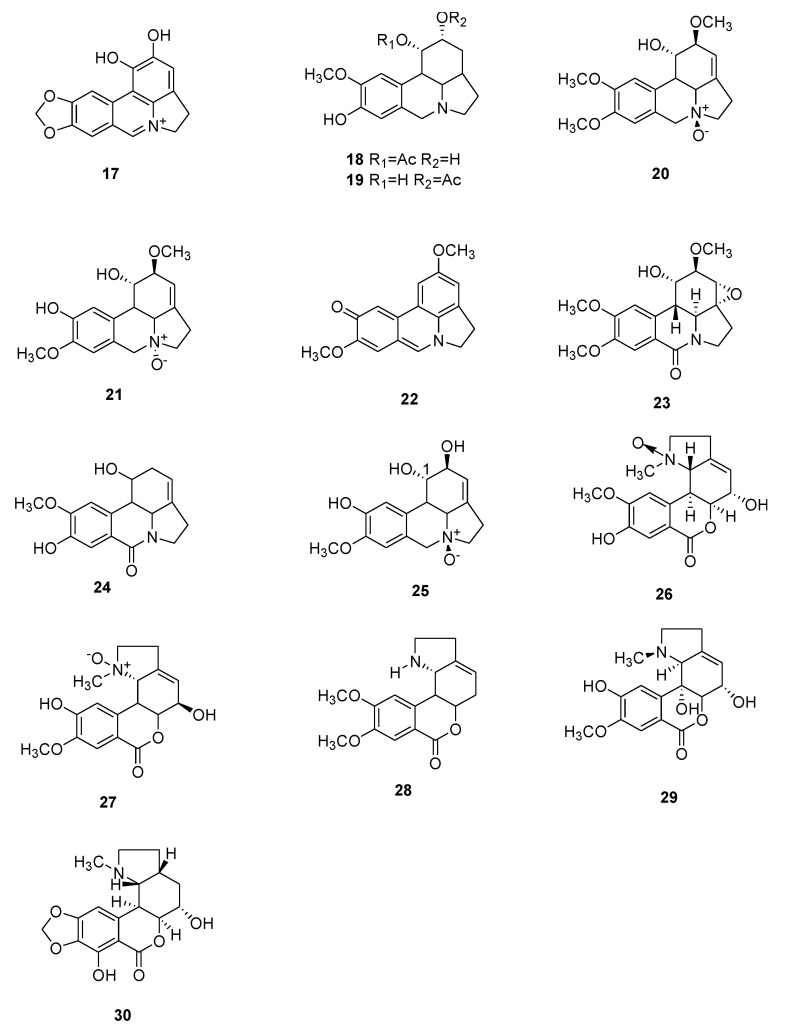
Chemical structure of new alkaloids of the lycorine- (**17**–**25**) and homolycorine-type (**26**–**30**). Numbers in bold refer to the compounds depicted in Table 2.

**Figure 5:**
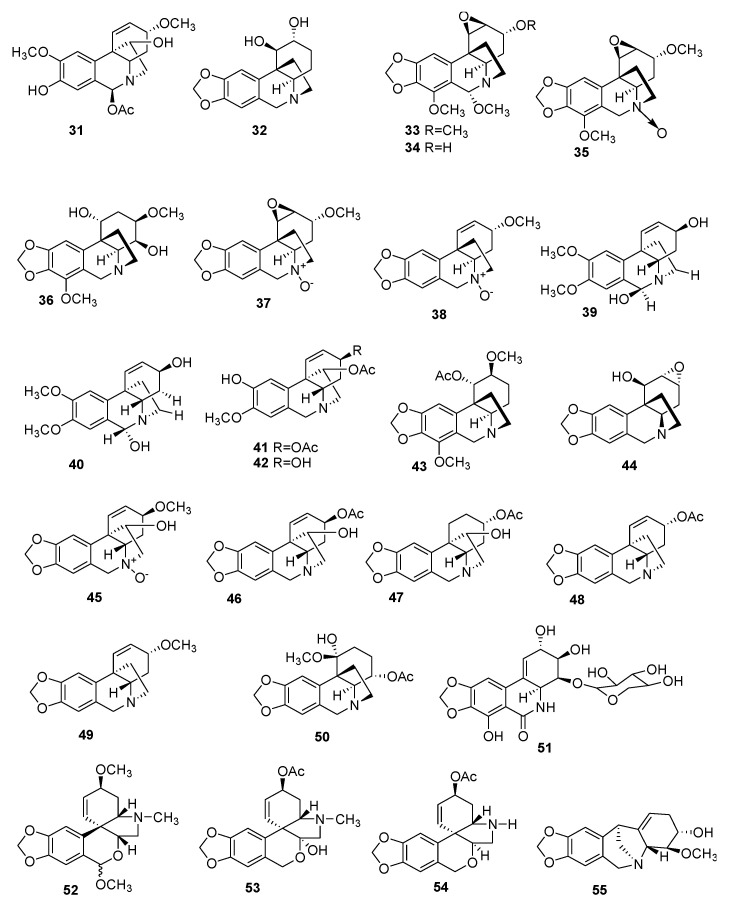
Chemical structure of new alkaloids crinine-, narciclasine-, tazettine-, and montanine-type (**31**–**55**). Numbers in bold refer to the compounds depicted in Table 2.

**Figure 6:**
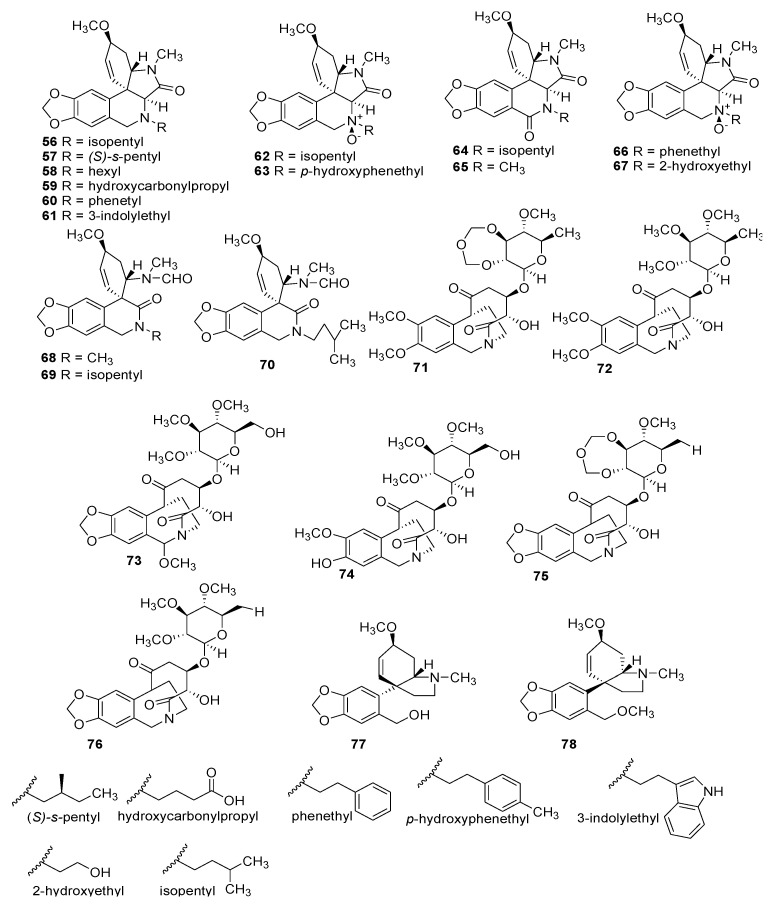
Chemical structure of new alkaloids plcamine-, *seco*-plicamine-, cripowellin-, and mesembrine-type. Numbers in bold refer to the compounds depicted in Table 2.

**Figure 7:**
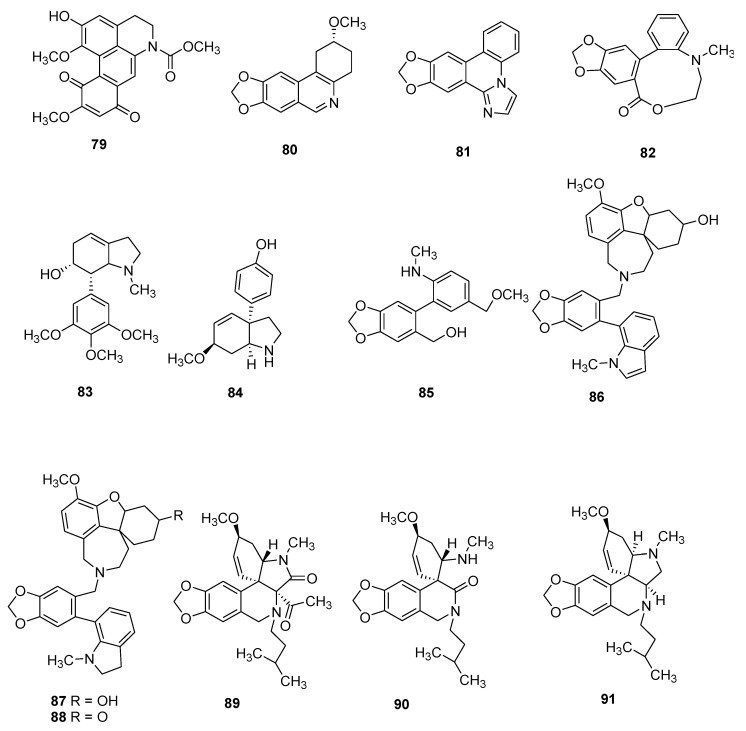
Chemical structure of new alkaloids of other types. Numbers in bold refer to the compounds depicted in Table 2.

Several of these novel AAs belong to known structural types, while others harbor new structures. The 91 AAs were classified in this manuscript as I) norbelladine-type (**1**–**6**), II) cherylline-type (**7**, **8**), III) galantamine-type (**9**–**16**), IV) lycorine-type (**17**–**25**), V) homolycorine-type (**26**–**30**), VI) crinine-type (**31**–**50**), VII) narciclasine-type (**51**), VIII) pretazettine-type (**52**–**54**), IX) montanine-type (**55**), and X) other types including AAs related to plicamine (**56**–**67**), *seco*-plicamine (**68**–**70**), cripowellin (**71**–**76**), mesembrine (**77**, **78**), and various others AAs (**79**–**91**). Structures of novel AAs belonging to the I to III scaffold types (norbelladine-, cherylline-, and galantamine-type) are depicted in Figure 3, whereas types IV and V (lycorine- and homolycorine-type) are represented in Figure 4. Figure 5 displays the structures of the para-para phenol coupled AAs of the VI to IX types including crinine-, narciclasine-, tazettine-, and montanine-type. AA of the type X including plicamine-, *seco*-plicamine, cripowellin, and mesembrine-types are represented in Figure 6, whereas all other types with different chemical structures are shown in Figure 7.

*Lycoris* genus encompasses 20 species originating from south and east Asia [90]. From the ethanol extract of the bulbs of *L. radiata*, two known alkaloids, (+)-*6*β-acetyl-crinamine and 8-demethyl-homolycorine-α-*N*-oxide, and four new alkaloids, namely (+)-1-hydroxy-ungeremine (**17**), (+)-6β-acetyl-8-hydroxy-9-methoxy-crinamine (**31**), (+)-2-hydroxy-8-demethyl-homolycorine-α-*N*-oxide (**26**) and (+)-*N*-methoxylcarbonyl-2-demethyl-isocorydione (**79**), belonging to the lycorine-, crinine-, homolycorine- and other-types respectively, were isolated. Their structural elucidation was performed by spectroscopic methods, such as 1D and 2D (^1^H–^1^H, COrrelation SpectroscopY (COSY), Heteronuclear Multiple Quantum Coherence (HMQC), and Heteronuclear Multiple Bond Correlation (HMBC)) Nuclear Magnetic Resonance (NMR) spectroscopy, in addition to high resolution mass spectrometry [67]. From the bulb of *L. radiata*, were also isolated four new AAs, lycoranines C-F (**9**, **27**, **28**, **80**), respectively belonging to galantamine- (**9**), homolycorine- (**27**, **28**) and other-types (**80**), with known alkaloids homolycorine, haemanthidine, haemanthamine, α-dihydrolycorine, galanthine, lycorine, and pseudolycorine. Their structure was determined using extensively NMR and high-resolution electrospray ionization mass spectrometry (HR-ESIMS) data. Their absolute and relative configuration were assigned by Electronic Circular Dichroism (ECD) and Rotating-frame Overhauser Spectroscopy (ROESY) NMR experiments [64].

The *Narcissus* genus is composed of approximately 50 species that originate from the area of the Iberian Peninsula. Jonquailine (**52**), a pretazettine-type AA, was isolated from the dried bulbs of *N. jonquilla quail* and characterized for structural elucidation and absolute configuration using various spectroscopic techniques [83]. Narcipavline (**6**) and narcikachnine (**87**), two new alkaloids, were isolated, together with thirteen known alkaloids, from fresh bulbs of *N. poeticus* cv. Pink Parasol, their chemical structure was elucidated by MS, together with 1D and 2D NMR spectroscopic analyses, and by comparison with literature data [29]. From the fresh bulbs of *N. pseudonarcissus* L. cv. Dutch Master, a new AA, named narcimatuline (**88**), was isolated, together with twenty-one known AAs of various structural types. Their chemical structure was elucidated by a combination of MS, HR-MS, 1D and 2D NMR spectroscopic techniques and also by comparison with existing data [89].

The phytochemical investigation of fresh bulbs of *Narcissus pseudonarcissus* cv. Carlton led to the isolation of thirteen known AAs, and three new norbelladine-type AAs: carltonine A–C (**4**–**6**). Their structure was determined using spectroscopic methods including 1D NMR, 2D NMR, and HR-MS [62]. 7-Oxonorpluviine (**24**), a lycorine-type alkaloid was isolated from fresh bulbs of *Narcissus* L. cv. Professor Einstein, together with twenty three known AAs, and their structures were identified by using various spectroscopic methods like (GC-MS, LC-MS, 1D, and 2D NMR spectroscopy) [71]. Pseudolycorine *N*-oxide (**25**) was isolated from *Narcissus tazetta* whole plant, and the structural elucidation was determined by spectroscopic data analysis [72].

Twenty species are included in the genus *Galanthus* native of Europe and of Middle East [91]. From whole plants of *Galanthus fosteri* Baker, three new AAs, namely oxoincartine (**23**), 3,11-*O*-diacetyl-9-*O*-demethylmaritidine (**41**) and 11-*O*-acetyl-9-*O*-demethylmaritidine (**42**) belonging to the lycorine and crinine-type, were isolated together with seven known compounds. Their structure was elucidated by spectroscopic analyses (UV, IR, MS, ECD, and 1D/2D NMR) [70].

*Hymenocallis* plants are native of Central and South America and include more than 60 species. Novel AA hymenolitatine (**82**) was isolated together with trispheridine and tazettine from the dichloromethane extract of the bulbs of *Hymenocallis littoralis* (Jacq.) Salisb. Chemical characterization of these compounds was performed by spectroscopic methods including 1D NMR, 2D NMR, and HR-MS [88].

The *Hippeastrum* genus contains approximately 94 species native of tropical and subtropical regions of the America [92]. One new alkaloid named 2α-10bα-dihydroxy-9-*O*-demethylhomolycorine (**29**), belonging to homolycorine-type and seven known alkaloids were isolated from the bulbs of *Hippeastrum solandriflorum*. Structural elucidation was performed by spectroscopic methods NMR (^1^H–^1^H, COSY, HMQC, HMBC, and Nuclear Overhauser Effect SpectroscopY (NOESY)) and mass spectrometry (HR-ESIMS) [73]. From a phytochemical study of the fresh bulbs and leaves of *Hippeastrum reticulatum*, eight known alkaloids and four new alkaloids named 6α-hydroxymaritidine (**39**), 6β-hydroxymaritidine (**40**), reticulinine (**18**) and isoreticulinine (**19**) were identified. The epimers (6α-hydroxymaritidine (**39**) and 6β-hydroxymaritidine (**0**)) were isolated as a mixture. Chemical characterization was performed by NMR, they belong to the crinine and lycorine-types [68]. Two novel AAs, 4-*O*-methylnangustine (**55**) and 7-hydroxyclivonine (**30**), of montanine and homolycorine types, respectively, and four known alkaloids were isolated from the bulbs of *H. argentinum* [74]. Phytochemical investigation of fresh bulbs and leaves of *Hippeastrum aulicum* (Ker Gawl.) Herb. enabled the characterization of the new alkaloid haemanthamine *N-*oxide (**45**), chemically characterized by NMR technics [79].

The *Nerine* genus contains 30 species that originate from South Africa and several may go extinct due to loss of habitat [93]. Two new AAs, 6-*O*-demethylbelladine (**1**) and 4′-*O*-demethylbelladine (**2**), belonging to the norbelladine-type, together with twenty known alkaloids, were isolated from fresh bulbs of *Nerine bowdenii* by standard chromatographic methods [60]. From the organic extract of *N. sarniensis*, an indigenous South African Amaryllidaceae, were isolated two new alkaloids named sarniensinol (**77**) and sarniensine (**78**) belonging to the mesembrine group, and a new crinine-type alkaloid named crinsarnine (**43**), together with bowdensine, tazettine, 3-epimacronine, lycorine, 1-*O*-acetyl-lycorine, and hippadine. Sarniensinol (**77**), sarniensine (**78**), and crinsarnine (**43**) were characterized by extensive spectroscopic and chiroptical methods [78,86].

The *Zephyranthes* genus encompasses more than 70 species native to the Americas. From the whole dried plants of *Zephyranthes candida*, Zephycandidine A (**81**), a first naturally occurring imidazol[1,2-f] phenanthridine alkaloid was isolated and characterized by spectroscopic analyses and NMR calculations [87]. From the 95% EtOH extract of the whole dried plant of *Z. candida*, three new alkaloids, zephycandidines I-III (**83**–**85**) were isolated, and their structures were elucidated by spectroscopic methods (HR-ESIMS, ^1^H NMR, ^13^C NMR, DEPT, ^1^H-^1^H COSY, HSQC, HMBC, ROESY). The absolute configuration of zephycandidines II and III (**84**–**85**) was estimated by ECD calculation, while the absolute configuration of zephycandidine I (**83**) was determined by single crystal X-ray diffraction using Cu Kα radiation [30]. Sixteen new alkaloids belonging to the galantamine (**11**−**16**), plicamine (**56**−**63**), and *seco*-plicamine (**68** and **69**) types, together with eight known compounds, were isolated from the whole plant of *Z. candida*. The chemical structures of these alkaloids were determined by extensive spectroscopic analyses, and absolute configurations of **11**, **12**, **56** and **57** were confirmed by single crystal X-ray diffraction analysis [66]. Further study on whole plants of *Z. carinata* led to the isolation of eleven new AAs, classified as 12-acetylplicamine (**89**), *N*-deformyl-*seco*-plicamine (**90**), plicamine (**64**−**67**), 4a-*epi*-plicamine (**1**), *seco*-plicamine (**70**), and lycorine (**20**−**22**) framework types, together with fifteen known alkaloids. The chemical structure of these alkaloids was determined by extensive spectroscopic analyses, and absolute configuration of **20** and **21** was confirmed by single crystal X-ray diffraction analysis. Zephycarinatines A (**74**), B (**75**), and G (**76**) represent the first examples of 12-acetylplicamine, *N*-deformyl-*seco*-plicamine, and 4a-*epi*-plicamine alkaloids, respectively [69]. Narciclasine-4-*O-*β*-*d-xylopyranoside (**51**), a new narciclasine glycoside, was isolated from the whole plant of *Zephyranthes minuta*. The structure of this new alkaloid was resolved using spectroscopic data analysis.

*Crinum* genus contains 180 species native of sub-Saharan and pantropical region. Four new potently bioactive cripowellin derivatives. 4,8-Dimethoxy-cripowellin C (**71**), 4,8-dimethoxy-cripowellin D (**72**), 9-methoxy-cripowellin B (**73**), and 4-methoxy-8-hydroxy-cripowellin B (**74**), together with one known alkaloid, cripowellin C were isolated from 95% EtOH extract of the bulbs of *Crinum latifolium*. Structural elucidation was performed by spectroscopic methods like NMR (^1^H–^1^H, COSY, HMQC, and HMBC) and mass spectrometry (HR-ESIMS) [84]. From the rhizome and fruits of *Crinum asiaticum* var. japonicum, two new AAs, crijaponine A (**32**) and crijaponine B (**10**) belonging to the crinine and galantamine-class, were isolated, together with eleven known alkaloids, ungeremine, lycorine, 2-*O*-acetyllycorine, 1, 2-*O*-diacetyllycorine, (−)crinine, 11-hydroxyvittatine, hamayne, (+)-epibuphanisine, crinamine, yemenine A, and epinorgalantamine. Structural elucidation was performed by spectroscopic methods including 1D and 2D NMR [65]. From the leaves of *C. latifolium*, three new crinine-category alkaloids, named 6-methoxyundulatine (33), 6-methoxycrinamidine (**34**), and undulatine *N*-oxide (**35**), were isolated, together with eight known alkaloids, 6-hydroxyundulatine, 6-hydroxybuphanidrine, undulatine, crinamidine, ambelline, filifoline, augustamine, and perlolyrine. Structural elucidation and absolute configuration were performed by various spectroscopic techniques such as NMR, ECD, and HR-Q-TOF-MS [75]. Antimalarial bioassay-guided fractionation MeOH extracts from dried powder of above-ground parts of *Crinum erubescens*, led to the isolation of two new alkaloids, cripowellin C (**75**) and cripowellin D (**76**), all together with two known alkaloids, cripowellin A and cripowellin B. Structural elucidation was performed using 1D and 2D-NMR techniques [85]. Phytochemical investigation of fresh bulbs and leaves of *Crinum amabile*, led to the identification of twenty-three alkaloids by GC-MS, and the isolation of two new crinine-kind alkaloids named augustine *N*-oxide (**37**) and buphanisine *N*-oxide (**38**). Their chemical structure was elucidated by NMR [77]. Three new cherylline- and crinine-type of AAs, named gigantelline (**7**), gigantellinine (**8**) and gigancrinine (**44**), were isolated from *Crinum jagus* (*Crinum giganteum*) collected in Senegal, together with the already known sanguinine, cherylline, lycorine, crinine, flexinine and the isoquinolinone derivative hippadine. All of the new isolated alkaloids were characterized by using spectroscopic (1D and 2D ^1^H and ^13^C NMR and HR-ESIMS) and chemical methods. Their relative configuration was assigned by NOESY NMR spectra and NMR calculations, while the absolute configuration was assigned using ECD experiments and calculations [63]. From bulbs of *Crinum asiaticum* L., two new alkaloids, crinasiaticine A (**46**) and crinasiaticine B (**47**), and fifteen known alkaloids were isolated. Their structural elucidation and absolute configuration were performed by spectroscopic methods (HR-TOF-MS, ^1^H NMR, ^13^C NMR, ^1^H-^1^H COSY, HMBC, NOESY, ECD) [80]. Four new alkaloids named, scillitazettine (**53**), scilli-*N-*desmethylpre*tazettine* (**54**), 3-*O-*acetylvittatine (**48**), and 4′-*O*,*N-*dimethylmethylnorbelladine *N-*oxide (**3**) were isolated from bulbs of *Crinum scillifolium*. Their structure was elucidated using various spectroscopic methods (1D and 2D ^1^H and ^13^C NMR and HR-ESIMS). Their relatives and absolute configurations were determined by DFT-NMR and ECD calculations [61].

*Amaryllis* is a small genus comprised of two species (*A. belladonna* L. and *A. paradisicola* Snijman). In particular *A. belladonna* is native of South Africa [94]. A new crinine alkaloid 1,4-dihydroxy-3-methoxy powellan (**36**), together with five known alkaloids, were isolated from the bulbs of *A. belladonna* Steud. by bioassay-guided isolation. Structures were elucidated by interpretation of combined HR-ESIMS, CD, and 2D NMR spectroscopic data [76].

*Brunsvigia* genus contains seventeen species endemic to Southern Africa [95]. Two new AAs named 3-*O*-methyl-*epi*-vittatine (**49**) and crouchinine (**50**) were isolated from bulbs of *Brunsvigia natalensis*. The AAs structures were determined by spectroscopic analyses (UV, FT-IR, MS, ECD, and 1D/2D NMR) [81].

## 5. Pharmacological Properties of Novel Amaryllidaceae Alkaloids

The pharmacological properties of the newly discovered AAs (**1**–**91**) were assessed when isolated in sufficient amount. AAs display a wide range of biological activities, including cytotoxicity, effects on the central nervous systems (CNS), anti-inflammatory, anti-microbial, anti-parasitic, larvicidal, and antioxidant activities.

### 5.1. Antitumoral Cytotoxic Activity

Lycorine is the most abundantly found AA, it belongs to the pyrrolo[*de*]phenanthridine subgroup. The biological effects of lycorine have been known for many years, and lycorine is still being investigated for a variety of therapeutic application, in particular as anticancer agent showing promising activity against tumors with dismal prognoses [96,97]. The structure–activity relationship (SAR) of lycorine and its derivatives has been evaluated using human leukemia T cells (Jurkat). The results showed that the free 1,2-diol functionality in the C-ring is required to induce apoptosis [98]. Furthermore, it has been demonstrated that the presence of the unaltered diol functionality in the C-ring in its original configuration in lycorine series, the stereochemistry of the C/D ring junction and the conformational freedom of the C-ring were required for the anticancer activity [96]. 4′-*O*,*N-*dimethylnorbelladine *N-*oxide (**3**) displayed a weak cytotoxicity activity against the human colon cancer cell line HCT116 at concentration ranging from 10^−5^ M and 10 ^−6^ M [61].

Among the new lycorine-type alkaloids, (+)-1-hydroxy-ungeremine (**17**) was evaluated for its cytotoxic potential against BEN-MEN-1 (meningioma), CCF-STTG1 (astrocytoma), CHG-5 (glioma), SHG-44 (glioma), U251 (glioma), HL-60 (human myeloid leukemia), SMMC-7721 (hepatocellular carcinoma), and W480 (colon cancer) cell lines and exhibited the most potent cytotoxicity against all tested tumor cell lines except BEN-MEN-1, with IC_50_ values ranging from 9.4 to 11.8 μM [67]. Pseudolycorine *N*-oxide (**25**) was inactive against human cervical cancer (SiHa) and human epidermoid carcinoma (KB) cells [72].

Among the homolycorine-type alkaloids, (+)-2-hydroxy-8-demethyl-homolycorine-α-*N*-oxide (**26**) had no significant cytotoxic activity (IC_50_ > 80 μM) against BEN-MEN-1 (meningioma), CCF-STTG1 (astrocytoma), CHG-5 (glioma), SHG-44 (glioma), U251 (glioma), HL-60 (human myeloid leukemia), SMMC-7721 (hepatocellular carcinoma), and W480 (colon cancer) cell lines [67]. Lycoranines E and F (**27** and **28**) showed moderate cytotoxicity against A549 (human lung carcinoma) and LoVo (human colon carcinoma) cells lines with IC_50_ > 20 μM [64]. 2α-10bα-Dihydroxy-9-*O*-demethylhomolycorine (**29**) showed significant cytotoxicity against the HCT-116 (colon adenocarcinoma), OVCAR-8 (ovarian carcinoma) and SF-295 (glioblastoma) cell lines with IC_50_ values 11.69, 15.11, and 16.31 μM respectively [73].

Numerous additional types of AAs displayed interesting anti-cancer activity. Of the cherylline-type, gigantellinine (**8**) had a weak but significant cytotoxicity at 400 μM against breast cancer cell line MCF-7, while gigantelline (**7**) showed no cytotoxicity at the same concentration [63]. Crinine-derivatives (+)-6β-acetyl-8-hydroxy-9-methoxy-crinamine (**31**) showed significant cytotoxicity against HL-60 (IC_50_ < 10 μM), and moderate cytotoxicity against astrocytoma and glioma cell lines, CCF-STTG1, CHG-5, SHG- 44 and U251 (10 μM < IC_50_ ≤ 30 μM) [67]. The cleavage between C-1 and C-13 and the hydroxyl at C-6′ in crinine alkaloid skeleton might be essential to their bioactivity [84]. Crijaponine A (**32**) was inactive towards both human epithelial carcinoma HeLa and HL-60 cell lines (IC_50_ > 60 μM) [65]. 6α-Methoxyundulatine (**33**), 6α-methoxycrinamidine (**34**), and undulatine *N*-oxide (**35**) did not show significant cytotoxicity against the KB (derived from a human carcinoma of the naropharynx), HepG2 (human liver cancer), MCF7 (breast cancer), SK-Mel2 (melanoma), and LNCaP (human prostate) cancer cell lines with IC_50_ > 100 μM [75]. 1,4-Dihydroxy-3-methoxy powellan (**36**) displayed an IC_50_ > 60 μM against the A2780 human ovarian cancer cell line [76]. Gigancrinine (**44**) showed no cytotoxicity at 400 μM against breast cancer cell line MCF-7 [63]. 3-*O*-Methyl-*epi*-vittatine (**49**) did not shown any cytotoxicity against MCF7, TK10 and UACC62 cancer cell lines [81]. Narciclasine-4-*O-*β*-*d-xylopyranoside (**51**), a narciclasine-type was inactive against KB and SiHa cell lines at all the concentrations [82]. Jonquailine (**52**), a pretazettine-tye, showed significant antiproliferative activities against cells derived from glioblastoma (U87, U373, and Hs683), melanoma (SKMEL-28), uterine sarcoma (MES-SA and MES-SA/Dx5), and lung carcinoma (A549, H1993 and H2073) with IC_50_ values ranging from 1 to 85 μM [83]. Moreover, (**52**) showed synergic effects with paclitaxel in its anti-proliferative action against lung carcimoma drug-resistant H1993 and H2073 cells in a dose-dependent manner with IC_50_ values between 0.39 and 100 μM. The SAR of (**52**) suggested that hydroxylation of C-8 was required for its anticancer activity [83].

Among cripowellin-type AAs, 4,8-dimethoxy-cripowellin C (**71**), 4,8-dimethoxy-cripowellin D (**72**), 9-methoxy-cripowellin B (**73**), and 4-methoxy-8-hydroxy-cripowellin B (**74**) showed impressive cytotoxicity against seven lung cancer cell lines (A549, H446, H460, H292, 95-D, and SPCA-1) with IC_50_ < 30 nM, with (**73**) and (**74**) being more active than (**71**) and (**72**). Cripowellin C (**75**) and cripowellin D (**76**) were efficiently cytotoxic against the A2780 human ovarian cancer cell line with IC_50_ values of 25 ± 2, and 28 ± 1 nM, respectively [85].

Other-type of AA (+)-*N*-methoxylcarbonyl-2-demethyl-isocorydione (**79**) exhibited strong cytotoxic against all tested tumor cell lines (astrocytoma, glioma, human myeloid leukemia, hepatocellular carcinoma, colon cancer) except meningioma (BEN-MEN-1), with IC_50_ values of 9.2–12.8 μM [67]. Zephycandidine A (**81**) was cytotoxic for five cancer cell lines, HL-60, A549, MCF-7, colon cancer SW480 (colon cancer), and hepatocellular carcinoma SMMC- 7721 (hepatocellular carcinoma), with IC_50_ values of 1.98, 6.49, 3.44, 6.27, and 7.03 μM, respectively. Moreover, zephycandidine A (**81**) showed weak cytotoxicity against the normal Beas-2B cell line (IC_50_ = 20.08 μM) with selectivity index as high as 10 when compared normal Beas-2B cell line, via activation of caspase-3, upregulation of Bax, down-regulation of Bcl-2, and degradation of PARP expression [87]. Hymenolitatine (**82**) showed weak cytotoxic activity against four cancer cell lines, HepG-2, LoVo, Hela, and A549, with IC_50_ values of 75.19, 69.81, 96.37, and 102.53 μM, respectively [88]. Cripowellin-form (**71**–**76**) and Zephycandidine A (**81**) belonging to the other-type of AA may be potential targets for further anticancer investigations.

### 5.2. Effects on the Central Nervous System (CNS)

Several enzymes of the CNS are interesting drug targets. AChE is a serine protease located at neuromuscular junctions, in cholinergic synapses of the central nervous system and in red blood cells [99,100,101]. The enzyme catalyzes the rapid hydrolysis and inactivation of the neurotransmitter acetylcholine into acetate and choline to enable cholinergic neurons to return to their resting state. Butyrylcholinesterase (BChE) can also hydrolyze acetylcholinesterase into acetate and choline. BChE is produced by the liver and detected in the plasma. Changes in its plasmatic levels can indicate of liver dysfunction. BChE is also expressed in neurons of the CNS [102].

In Alzheimer’s disease (AD), AChE is overly active, and the consequential lower level of acetylcholine in the brain cause weakened neurotransmission [103]. Similarly, BChE deregulation is measured in the brain of individuals suffering from AD. Malfunction of the cholinergic system may be pharmacologically tackled via AChE inhibitors that ameliorate the cholinergic deficit at early stages of the disease and reduce progression. In addition, glycogen synthase kinase-3 (GSK-3) is a ubiquitous serine/threonine kinase, implicated in AD, which can trigger abnormal hyperphosphorylation of tau protein, which is believed to be a critical event in neurofibrilary tangle formation. Thus, GSK-3 inhibition represents an attractive drug target for AD and other neurodegenerative disorders [104]. Finally, prolyloligopeptidase (POP) is a cytosolic serine peptidase widely distributed in the organs of the body, including the brain, which cleaves peptide bonds at the carboxyl end of proline [105,106]. Previous studies have shown that POP inhibitors are efficient anti-dementia drugs [107,108].

The AA galantamine, donepezil and rivastigmine are potent reversible inhibitors of AChE approved for the symptomatic treatment of AD [109,110]. Since cholinesterase enzyme inhibitors are first generation drugs for AD, AChE and BChE are the most targeted enzymes at the moment.

Galantamine derivative sanguinine is ten times more active than galantamine whereas 11-hydroxygalantamine exhibits inhibitory activity similar to that of galantamine. The extra or protected hydroxyl group in its allylic position in (R_1_) may be required for the activities [111]. SAR of galantamine and its derivatives was comprehensively reviewed elsewhere [8].

Among the six new galantamine-type alkaloids only 9-de-*O*-methyl-*11*β-hydroxygalantamine (**13**) showed a weak AChE inhibitory activity with IC_50_ value 168.7 μM. The SAR of new galantamine derivatives alkaloids (**11**–**16**) and known alkaloids isolated from the same plant species revealed that the 4,4a double bond and 9-OH are required for the AChE inhibitory activity, while the presence of the 11-OH group dramatically decreases AChE inhibitory activity [66].

Norbelladine-type alkaloids 6-*O*-demethylbelladine (**1**) and 4′-*O*-demethylbelladine (**2**) were identified as weak inhibitors of AChE with IC_50_ values of 223.2 ± 23.6 and 606.8 ± 74.2 μM, respectively, for (**1**) and (**2**) [60]. They were more potent against BChE, (**2**) (IC_50_ = 30.7 ± 4.0 μM) being more active than (**1**) (IC_50_ = 115.7 ± 10.1μM). 4′-*O*-Demethylbelladine (**2**) exhibited weak POP inhibition with IC_50_ value of 370 ± 30 μM), but more potently than 6-*O*-demethylbelladine (**1**) (IC_50_ = 660 ± 90 μM) [60]. Carltonine A–C (**4**–**6**) were evaluated for their potential activity against AChE, BChE and POP. Carltonine A–B (**4** and **5**) inhibited BChE with IC_50_ values of 0.91 ± 0.02 and 0.031 ± 0.001 μM. Computational studies detected a plausible binding site on BChE while SAR suggested that the 1,3-dioxolane ring of carltonine B of (**5**) may be responsible of the BChE activity compared to the opening dimethoxybenzene analogue of (**4**) [62].

As for the cherylline-type, gigantellinine (**8**) inhibited the activity of AChE in a dose-dependent manner with IC_50_ value of 174.90 ± 2.30 μM, while gigantelline (**7**) did not [63].

The lycorine-type, galanthine *N*-β-oxide (20) did not exhibit significantly AChE inhibitory activity (IC_50_ > 200 μM) compared to galanthine. The *N*-oxide fragment seems to be deactivating the inhibitory activity of AChE. New lycorine derivatives (**22**) displayed moderate AChE inhibition (IC_50_ = 35.61 ± 1.90 μM) compared to carinatine (IC_50_ > 200 μM). The aromatic C-ring in lycorine-sort alkaloids may be essential for their activity against AChE [69]. Oxoincartine (**23**) was inactive against both cholinesterases (AChE and BChE) [70].

7-Hydroxyclivonine (**30**), a homolycorine-type exhibited weak AChE inhibition with IC_50_ value 114.07 μM and moderate effect against BChE with IC_50_ = 67.3 μM. Molecular docking with BChE revealed that (**30**) and galantamine both interact with similar amino acids in the same binding pocket [74].

In the crinine subgroup, augustine *N*-oxide (**37**) showed moderate inhibition of AChE at 79.64 μg·mL^−1^ (250.44 μM), while buphanisine *N-*oxide (**38**) did not inhibit the enzyme activity [77]. A mixture of 6α-hydroxymaritidine (**39**) and 6β-hydroxymaritidine (**40**) showed weak AChE inhibitory activity with IC_50_ = 90.43 μM, and was inactive of for BChE inhibitory activity (IC_50_ > 600 μM). Molecular docking studies of 6α-hydroxymaritidine (**39**), 6β-hydroxymaritidine (**40**), lycorine-type reticulinine (**18**), and isoreticulinine (**19**) at the active sites of AChE and BChE identified (19) as a potential inhibitory molecule, since it was stabilized in the active site through hydrogen bonds, π-π stacking and hydrophobic interactions [68]. Crijaponine A (**32**) showed anti-AChE activity with IC_50_ > 10 μM [65]. Moreover, 11-*O*-Acetyl-9-*O*-demethylmaritidine (**42**) inhibited both cholinesterases (AChE and BChE) with IC_50_ values 6.04 and 29.72 μM, respectively. Furthermore, 3,11-*O*-Diacetyl-9-*O*-demethylmaritidine (**41**) showed weaker AChE inhibition with IC_50_ value 67.4 μM and was inactive against BChE. The SAR of some haemanthamine-derivatives revealed that two free hydroxyl groups present at C-3 and C-11 may be essential for the anti-cholinesterase activity [70]. Gigancrinine (**44**) did not show any anti-AChE activity [63].

The 4-*O*-Methylnangustine (**55**), a montanine-type AA was inactive (IC_50_ > 200 μM) against both AChE and BChE [74].

Among plicamine- and *seco-*plicamine subgroups of alkaloids, (**61**–**63**) exhibited weak AChE inhibition, with IC_50_ values of 110.6, 57.26, and 75.3 μM [66]. Moreover, (**67**) showed weak AChE inhibitory activity with IC_50_ value 126.16 μM, whereas the others new plicamine- and *seco*-plicamine-types were almost inactive (IC_50_ > 200 μM). The *N*-2-hydroxyethyl group in plicamine-form alkaloids may be essential for this activity [69].

Other types of alkaloids, zephycandidine III (**85**) significantly inhibited AChE with IC_50_ value of 8.82 μM, while (**83**) and (**84**) were inactive at 200 μM. Narcipavline (**86**) exhibited weak AChE inhibitory activity with IC_50_ value 208 ± 37 μM and a significant BChE inhibitory activity IC_50_ = 24.4 ± 1.2 μM. Zephycandidine A (**81**) displayed anti-AChE activity in a dose-dependent manner with IC_50_ = 127.99 μM. Molecular docking studies of zephycandidines (**81**), (**83**–**85**) and galantamine with AChE revealed that interactions with W286 and Y337 are necessary for inhibitory activity [30] [87]. Narcimatuline (**88**) was evaluated for its AChE, BChE, POP, and GSK-3β inhibitory activities and significant inhibited BChE, POP and GSK-3 activities, with IC_50_ values of 5.9 ± 0.2, 29.2 ± 1.0 and 20.7 ± 2.4 μM, respectively, but only a weak activity against AChE with IC_50_ value 489 ± 60 μM [89].

Of all the candidates investigated recently, crinine-derivative 11-*O*-acetyl-9-*O*-demethylmaritidine (**42**) and other-type Zephycandidine III (**85**) are interesting targets for further anti-AChE investigation for Alzheimer’s disease.

### 5.3. Anti-Inflammatory and Antioxidant Activity

Unfortunately, the anti-inflammatory activity of AAs is rarely reported. In vitro anti-inflammatory studies of fifteen AAs isolated from different *Crinum* species was comprehensively reviewed in 2003 and their activity was very low [112]. More recently, the anti-inflammatory activity of lycorine-type AAs, such as (+)-1-hydroxy-ungeremine (**17**), homolycorine-type, such as (+)-2-hydroxy-8-demethyl-homolycorine-α-*N*-oxide (**26**), crinine-type (+)-6β-acetyl-8-hydroxy-9-methoxy-crinamine (**31**), and other-type (+)-*N*-methoxylcarbonyl-2-demethyl-isocorydione (**79**) against cyclooxygenase-1 (COX,-1), and cyclooxygenase-2 (COX-2) was evaluated in vitro, and AA (**17**) and (**79**) displayed selective inhibition of COX-2 (>90%) [67].

Cripowellin derivatives, 4,8-dimethoxy-cripowellin C (**71**), 4,8-dimethoxy-cripowellin D (**72**), 9-methoxy-cripowellin B (**73**) and 4-methoxy-8-hydroxy-cripowellin B (**74**) displayed significant inhibition of COX-1 (>64%) and of COX-2 (>90%), respectively [84]. They were also evaluated for their antioxidant potential activities using ABTS^·+^ (2,2′-azino-bis(3-ethylbenzo-thiazoline-6-sulphonic acid) and DPPH (1,1-diphenyl-2-picrylhydrazyl) methods. AAs (**72**), (**73**), and (**74**) showed significant antiradical activity with IC_50_ values of from 52.2 to 80.1μM.

The anti-inflammatory activity of galantamine-, plicamin- and *seco*-plicamin-type (**11**−**16**, **56**–**67** and **68**–**69**) was evaluated in vitro by studying the inhibition of lipopolysaccharide (LPS)-induced nitric oxide (NO) production in RAW 264.7 mouse macrophages. Among the tested compounds, two plicamine-type alkaloids (**59**) and (**60**) showed significant inhibitory activities with IC_50_ values of 18.77 and 10.21 μM, respectively, while other alkaloids were inactive at 200 μM [66].

### 5.4. Anti-Parasitic and Antibacterial Activity

The 4,8-Dimethoxy-cripowellin C (**71**), 4,8-dimethoxy-cripowellin D (**72**), 9-methoxy-cripowellin B (**73**), and 4-methoxy-8-hydroxy-cripowellin B (**74**) were evaluated for their antimicrobial against eight species of bacteria (*Streptococcus pneumoniae*, *Staphylococcus aureus*, *Staphylococcus epidermidis*, *Klebsiella pneumoniae*, *Pseudomonas aeruginosa*, *Haemophilus influenzae*, *Enterobacter cloacae*, and *Shigella dysenteriae*). Moreover, (**73**) and (**74**) displayed highest antibacterial activity with IC_50_ values < 0.50 mM, while (**71**) and (**72**) had weak activity [84].

Malaria (*Plasmodium* sp.), leishmaniasis (*Leishmania* sp.), and trypanosomiasis (*Trypanosoma brucei* and *Trypanosoma cruzi*) are the most common chronic protozoan diseases and occur mainly in poor rural and urban areas in tropical and subtropical regions of the world. Previously, several AAs were reported for their potent in vitro antiprotozoal activity [113]. The anti-plasmodial activity was recently reviewed elsewhere [114,115]. Newly isolated alkaloids such as cripowellin C (**75**) and D (**76**) were evaluated against the chloroquine/mefloquine-resistant Dd2 strain of *Plasmodium falciparum* and were found to have potent antiplasmodial activity, with IC_50_ values of 180 ± 20, 26 ± 2, and 260 ± 20 nM, respectively [85].

Crinine-type 1,4-dihydroxy-3-methoxy powellan (**36**) had a weak activity, with IC_50_ = 37 ± 3 μM against the same pathogen [76]. The two epimers (6α-hydroxymaritidine (**39**) and 6β-hydroxymaritidine (**40**)) were evaluated for their antiprotozoal activity against *T. brucei rhodesiense* (trypomastigotes forms, STIB 900 strain), *T. cruzi* (amastigotes forms, Tulahuen C4 strain), *L. donovani* (amastigotes forms, MHOM-ET-67/L82 strain), and *P. falciparum* (intraerythrocytic forms, IEF, NF54 strain). They displayed low toxicity against all protozoans tested with IC_50_ values of 30.68, 66.11, > 100 and 32.86 μg.mL^−1^ respectively [68]. Augustine *N*-oxide (**37**) and buphanisine *N*-oxide (**38**) were also evaluated against the same strains mentioned above and displayed low activity with IC_50_ values ranging from 32 to > 100 μg.mL^−1^. The presence of an *N*-oxide group in (**37**) and (**38**) appears to decrease their activity against *T. brucei* and *P. falciparum* compared to the previously characterized anti-protozoal compounds belonging to the same subgroup [77].

Scillitazettine (**53**) and scilli-*N-*desmethylpre*tazettine* (**54**) were evaluated against the chloroquine-resistant strain *P. falciparum* FcB1 and displayed antiparasitic activity with IC_50_ values of 77.0 ± 2.0 and 46.5 ± 2.0 μM, respectively [61].

### 5.5. Larvicidal and Insecticidal

Insects are important vectors of many diseases, controlling their proliferation is an efficient way of preventing disease spread. *Aedes aegypti* is the main vector for dengue, yellow fever and Zika infection. In an earlier study, organic extracts of the bulbs of *Nerine sarniensis*, demonstrated strong larvicidal and insecticidal activity with LC_50_ of 0.008 μg.μL^−1^ against *A. aegypti* larvae and against grown-up females with LD_50_ of 4.6 μg/mosquito. Sarniensine (**78**) was less efficient against larvae at the most minimal concentration of 0.1 μg/μL but displayed strong adulticidal activity with an LD_50_ of 1.38 ± 0.056 μg/mosquito [86]. Mesembrine-class sarniensinol (**77**), sarniensine (**78**) and crinine-type crinsarnine (**43**) had no effect against *A. aegypti* larvae at all concentrations tested. In adult topical bioassays, only (**43**) displayed adulticidal activity, with an LD_50_ = 2.29 ± 0.049 μg per mosquito. SAR studies revealed that the scaffold of pretazettine alkaloids in (**77**) and (**78**) and (**43**) and in bowdensine were important for their activity. Among the mesembrine group, the opening of the B-ring or the presence of a B-ring lactone as well as the trans-stereochemistry of the A/B-ring junction seem to be important for their activity, while in crinine-type alkaloids, the substituent at C-2 appears to be important [78,86,116].

### 5.6. Others Activities

The carbonic anhydrases (CAs) are metalloenzymes that catalyze the reversible hydration of carbon dioxide with water into a bicarbonate ion and a proton. In humans, sixteen isozymes have been identified including human cabonic isozyme II (hCAII) reported to be involved many diseases like glaucoma, epilepsy and cancer. Thus, hCAII is a target for therapeutic interventions [80]. Crinasiaticine A (**46**) and crinasiaticine B (**47**) were evaluated for their inhibitory potential against hCAII and they were inactive [80].

## 6. Production of Amaryllidaceae Alkaloids

### 6.1. Chemical Extraction from Amaryllidaceae Plants

The acid-base extraction is the classical method used to extract alkaloids from Amaryllidaceae. The acid extraction usually uses 0.1% to 2% sulfuric acid (H_2_SO_4_) or hydrochloric acid (HCl) as solvent by maceration. For example, in a recent study by our group, fresh bulbs of *C. jagus* were dried at room temperature and then finely powdered [63]. The resultant powder (1.35 kg) was extracted with 1% H_2_SO_4_, (2 × 2 L) overnight at room temperature. The suspension was filtered through a cloth and successively centrifuged at 10 °C at 7000 rpm for 30 min. The acid extract was alkalinized to pH 9–10 with 12 N NaOH. The aqueous solution was extracted with EtOAc (3 × 1.2 L), and the organic extracts were combined, dried (Na_2_SO_4_) and evaporated under reduced pressure to give a brown oil residue (3.0 g) [63].

Alcoholic solvent such as methanol or ethanol can also be used to extract both free and salt of alkaloids. For example, fresh bulbs (1.5 kg) and leaves (400 g) of *H. reticulatum* were collected, crushed and extracted with MeOH (2 × 1.0 L) at room temperature for 4 days, and the combined macerate was filtered and evaporated under reduced pressure. The crude extracts of the bulbs and leaves (117.3 and 53.5 g, respectively) were acidified with H_2_SO_4_ (2%, *v*/*v*) to pH 3 and extracted with Et_2_O (10 × 150 mL) and EtOAc (3 × 150 mL) to remove the neutral material. The aqueous solutions were basified with ammonia (25%, *v*/*v*) up to pH 9–10 and extracted with n-Hex (16 × 150 mL) to give the n-Hex extracts (0.14 and 0.02 g, respectively. This was followed by extraction with EtOAc (15 × 150 mL) to give the EtOAc extracts (1.6 and 0.3 g, respectively) and finally extracted with EtOAc:MeOH (3:1, *v*/*v*) (4 × 150 mL) to provide the EtOAc:MeOH extracts (2.10 and 1.56 g, respectively) [68].

### 6.2. Biotechnological Production of Amaryllidaceae Alkaloids

Usually, extraction of bioactive compound from its natural source is low, time-consuming and costly, which hinders further research and application. With the commercialization of galantamine as a drug, its demand from generic pharmaceutical companies increased, and production from native source became challenging to fulfill [117]. The diversity and quantity of AAs in plants also vary with plant species, developmental stage and types of tissue/cell. Plants growth can be affected by seasonal changes and environmental factors [25,42,118]. Therefore, mass production of AAs by cultivation of plants is not always sustainable.

Alternatively, several studies have depicted other approaches to produce specialized metabolites such as alkaloids by using in vitro culture of plant parts/tissues. Results from in vitro culture of *Narcissus confusus* and *Leucojum aestivum* show that alkaloids’ amounts are low in undifferentiated cells as compared to differentiated cells, indicating that undifferentiated cells are unsuitable for such biotechnological process [119,120,121]. Growth and production of alkaloids and biomass in in vitro system often vary with source and concentration of carbohydrate (sucrose, fructose, glucose etc.), phosphorous and nitrogen. Optimization of these basal requirements in growth medium is crucial and can be achieved by using mathematical analysis to enhance production of target metabolites, increase biomass, or both. Another important factor that can regulate phytochemical profile of culture cells are phytohormones, i.e., auxins and cytokinins. Optimal ratio of phytohormones is essential and specific to maintain different stages of cultured cells, which ultimately can result in variation of phytochemicals [54,122,123]. Biosynthesis of galantamine was not detected in the in vitro culture of *N. pseudonarcissus* cv. Carlton in absence of phytohormone, whereas high amounts of galantamine were detected in differentiated tissues cultured in low auxin (4 mg/L of NAA) as compared to undifferentiated tissue cultured in high auxin (20 mg/L of NAA) [54]. Furthermore, in nature it was observed that plants synthesize alkaloids in response to different biotic and abiotic factors [124]. Therefore, these factors, or the associated signaling compounds produced in response to these factors, can be used as elicitors to enhance the biosynthesis of specialized metabolites in in vitro techniques [125,126]. For example, using methyl jasmonate as an elicitor in Leucojum shoot culture can enhance production of galantamine by two-fold [127]. Physical parameters further modulate production in in vitro system. Light, temperature and bioreactor types can greatly enhance the production of AAs in in vitro system. Finally, with a better understanding of the effect of the environment, and of microorganism–plant interactions, applying this knowledge to artificial culture system could boost the production of AAs and should be considered as a promising approach.

Like other specialized metabolites, AAs can be produced by heterologous expression of genes cluster that express the enzymes required for their biosynthesis. Precise and reliable construction of DNA fragments can be rapidly achieved by using modern recombination and DNA assembly techniques. Several strategies have been developed to engineer microbial hosts, such as bacteria, or yeast, for the heterologous production of alkaloids and their precursors [128,129,130,131,132,133,134]. Synthetic biology approaches for production of plant-derived specialized metabolites by metabolic engineering have been carried out primarily in yeast (*Saccharomyces cerevisiae*) so far and to a lesser extent in *Escherichia coli*; whereas tobacco species *Nicotiana tabacum* and *Nicotiana benthamiana* have emerged as hosts for the heterologous expression of biosynthetic genes and production of specialized metabolites in plants [135,136,137,138]. However, the lack of knowledge in biosynthetic pathway hinders this approach as of yet. Elucidation of the reactions will be achieved by using modern sequencing technology interrelated with metabolite studies, systems biology, and bioinformatic analysis. This will not only provide techniques to produce AAs but also help with biosynthesis of novel AAs derivatives.

## 7. Conclusions

A total of 91 new AAs have been isolated in the last 5 years, and some of their biological activities have been uncovered. AAs are a rich group of specialized metabolites with pleiotropic effects that represent an important resource for new drugs discovery. They should be deeper exploited, at the image of galantamine used to treat the symptoms of AD. Recent years of AAs research have been marked by the discovery of compounds with potent anticancer (e.g., **17**, **52**, **72**–**74**, **75**, **76**, and **79**), anticholinesterase (**42** and **85**), anti-inflammatory (**59**, **60**, and **71**–**74**), antiparasitic (**75** and **76**), larvicidal and insecticidal (**43** and **77**), antibacterial and antioxidant (**73** and **74**) biological activities. This review helps expand the chemical library of AAs and their possible application in medicine.

## Figures and Tables

**Table 1:** Main types of Amaryllidaceae alkaloids grouped according to their ring type and biosynthetic origin.

Number	Type Name	Ring-Type
I	Norbelladine	*N*-(3,4-Dioxybenzyl)-4-oxyphenethylamine
II	Cherylline	Tetrahydroisoquinoline
III	Galantamine	6H-Benzof,f]-2-benzazepine
IV	Lycorine	Pyrrolo[d,e]phenanthridine
V	Homolycorine	2-Benzopyrano-[3,4-g]indole
VI	Crinine	5,10b-Ethanophenanthridine
VII	Narciclasine	Lycoricidine
VIII	Pretazettine	2-Benzopyrano [3,4-c]indole
IX	Montanine	5,11-Methanomorphanthridine
X	Other	Different ring types and biogenetic origin

**Table 2:** Novel Amaryllidaceae alkaloids. Name, activity, chemical formula, and anatomic origin are displayed. The bold number in the first column corresponds to the assigned number of its corresponding chemical structure shown in Figure 3, Figure 4, Figure 5, Figure 6 and Figure 7.

No	Alkaloid	Activity	Formula	Organ	Ref.
**I—NORBELLADINE-TYPE**					
**1**	6-*O*-Demethylbelladine	CNS	C_18_H_23_NO_3_	B	[60]
**2**	4′-*O*-Demethylbelladine	CNS	C_18_H_23_NO_3_	B	[60]
**3**	4′-*O*,*N-*dimethylnorbelladine *N-*oxide	Tum	C_17_H_22_NO_4_	B	[61]
**4**	Carltonine A	CNS	C_27_H_32_N_2_O_3_	B	[62]
**5**	Carltonine B	CNS	C_26_H_28_N_2_O_3_	B	[62]
**6**	Carltonine C	CNS	C_44_H_49_N_3_O_5_	B	[62]
**II—CHERYLLINE-TYPE**					
**7**	Gigantelline	CNS, Tum	C_18_H_21_NO_3_	B	[63]
**8**	Gigantellinine	CNS, Tum	C_18_H_21_NO_4_	B	[63]
**III—GALANTAMINE-TYPE**					
**9**	Lycoranine C	Tum	C_16_H_21_NO_3_	B	[64]
**10**	Crijaponine B	CNS, Tum	C_19_H_23_NO_5_	R, F	[65]
**11**	11β-Hydroxylycoramine	Inf	C_17_H_23_NO_4_	B, L, F	[66]
**12**	9-De-*O*-methyl-11β-hydroxylycoramine	Inf	C_16_H_21_NO_4_	B, L, F	[66]
**13**	9-De-*O*-methyl-11β-hydroxygalantamine	CNS, Inf	C_16_H_19_NO_4_	B, L, F	[66]
**14**	11β-Hydroxylycoramine *N*-oxide	Inf	C_17_H_23_NO_5_	B, L, F	[66]
**15**	11β-Hydroxygalantamine *N*-oxide	Inf	C_17_H_21_NO_5_	B, L, F	[66]
**16**	2β,11β-Dihydroxygalantamine	Inf	C_17_H_21_NO_5_	B, L, F	[66]
**IV—LYCORINE-TYPE**					
**17**	(+)-1-Hydroxy-ungeremine	Inf, Tum	C_16_H_12_NO_4_^+^	B	[67]
**18**	Reticulinine	CNS	C_17_H_21_NO_4_	B, L	[68]
**19**	Isoreticulinine	CNS	C_17_H_21_NO_4_	B, L	[68]
**20**	Galanthine *N*-β-oxide	CNS	C_18_H_23_NO_5_	B, L, F	[69]
**21**	Carinatine *N*-α-oxide	CNS	C_17_H_21_NO_5_	B, L, F	[69]
**22**	Zephycarinatine I	CNS	C_17_H_15_NO_3_	B, L, F	[69]
**23**	Oxoincartine	CNS	C_18_H_21_NO_6_	B, L, F	[70]
**24**	7-Oxonorpluviine	nm	C_16_H_17_NO_4_	B	[71]
**25**	pseudolycorine *N*-oxide	Tum	C_16_H_19_NO_5_	B, L, F	[72]
**V—HOMOLYCORINE-TYPE**					
**26**	(+)-2-Hydroxy-8-demethyl-homolycorine-α-*N*-oxide	Inf, Tum	C_17_H_19_NO_6_	B	[67]
**27**	Lycoranine E	Tum	C_17_H_19_NO_6_	B	[64]
**28**	Lycoranine F	Tum	C_17_H_19_NO_4_	B	[64]
**29**	2α-10bα-Dihydroxy-9-*O*-demethylhomolycorine	Tum	C_17_H_19_NO_6_	B	[73]
**30**	7-Hydroxyclivonine	CNS	C_17_H_19_NO_6_	B	[74]
**VI—CRININE-TYPE**					
**31**	(+)-6β-Acetyl-8-hydroxy-9-methoxy-crinamine	Inf, Tum	C_19_H_23_NO_6_	B	[67]
**32**	Crijaponine A	CNS, Tum	C_16_ H_19_ NO_4_	R, F	[65]
**33**	6α-Methoxyundulatine	Tum	C_19_H_23_NO_6_	L	[75]
**34**	6α-Methoxycrinamidine	Tum	C_18_H_21_NO_6_	L	[75]
**35**	Undulatine *N*-oxide	Tum	C_18_H_21_NO_6_	L	[75]
**36**	1,4-Dihydroxy-3-methoxy powellan	Par, Tum	C_18_H_23_NO_6_	B	[76]
**37**	Augustine *N*-oxide	CNS, Par	C_17_ H_19_NO_5_	B, L	[77]
**38**	Buphanisine *N*-oxide	CNS, Par	C_17_ H_19_NO_4_	B, L	[77]
**39**	6α-Hydroxymaritidine	CNS, Par	C_17_H_21_NO_4_	B, L	[68]
**40**	6β-Hydroxymaritidine	CNS, Par	C_17_H_21_NO_4_	B, L	[68]
**41**	3,11-*O*-Diacetyl-9-*O*-demethylmaritidine	CNS	C_20_H_22_NO_6_	B, L, F	[70]
**42**	11-*O*-Acetyl-9-*O*-demethylmaritidine	CNS	C_18_H_20_NO_5_	B, L, F	[70]
**43**	Crinsarnine	Ins, Lar	C_20_H_25_NO_6_	B	[78]
**44**	Gigancrinine	CNS, Tum	C_16_H_17_NO_4_	B	[63]
**45**	Haemanthamine *N-*oxide	nm	C_17_H_19_NO_5_	B, L	[79]
**46**	Crinasiaticine A	hCAII	C_18_H_19_NO_5_	B	[80]
**47**	Crinasiaticine B	hCAII	C_18_H_21_NO_5_	B	[80]
**48**	3-*O-*Acetylvittatine	Tum	C_18_H_19_NO_4_	B	[61]
**49**	3-*O*-Methyl-*epi*-vittatine	Tum	C_17_H_19_NO_3_	B	[81]
**50**	Crouchinine	nm	C_19_H_23_NO_6_	B	[81]
**VIII—NARCICLASINE-TYPE**					
**51**	Narciclasine-4-*O-*β*-*d-xylopyranoside	Tum	C_19_H_21_NO_11_	B, L, F	[82]
**VIII—TAZETTINE-TYPE**					
**52**	Jonquailine	Tum	C_19_H_23_NO_5_	B	[83]
**53**	Scillitazettine	Par, Tum	C_19_H_21_NO_6_	B	[61]
**54**	Scilli-*N*-desmethylpretazettine	Par, Tum	C_18_H_19_NO_5_	B	[61]
**IX—MONTANINE-TYPE**					
**55**	4-*O*-Methylnangustine	CNS	C_17_H_19_NO_4_	B	[74]
**X—OTHER-TYPES**					
**PLICAMINE**					
**56**	*N*-Isopentyl-5,6-dihydroplicane	Inf	C_23_H_30_N_2_O_4_	B, L, F	[66]
**57**	*N*-(*S*)-*s*-Pentyl-5,6-dihydroplicane	Inf	C_23_H_30_N_2_O_4_	B, L, F	[66]
**58**	*N*-Hexyl-5,6-dihydroplicane	Inf	C_24_H_32_N_2_O_4_	B, L, F	[66]
**59**	*N*-Hydroxycarbonylpropyl-5,6-dihydroplicane	Inf	C_22_H_26_N_2_O_6_	B, L, F	[66]
**60**	*N-*Phenethyl-5,6-dihydroplicane	Inf	C_26_H_28_N_2_O_4_	B, L, F	[66]
**61**	*N*-3-Indolylethyl-5,6-dihydroplicane	CNS, Inf	C_28_H_29_N_3_O_4_	B, L, F	[66]
**62**	*N*-Isopentyl-5,6-dihydroplicane *N*-oxide	Inf	C_23_H_30_N_2_O_5_	B, L, F	[66]
**63**	Bliquine *N*-oxide	CNS	C_26_H_28_N_2_O_6_	B, L, F	[66]
**64**	Zephycarinatine C	Inf	C_23_H_28_N_2_O_5_	B, L, F	[69]
**65**	Zephycarinatine D	Inf	C_19_H_20_N_2_O_5_	B, L, F	[69]
**66**	Zephycarinatine E	Inf	C_23_H_28_N_2_O_5_	B, L, F	[69]
**67**	Zephycarinatine F	CNS, Inf	C_20_H_24_N_2_O_6_	B, L, F	[69]
***SECO*-PLICAMINE**					
**68**	*N*-Methyl-11,12-*seco*-5,6-dihydroplicane	Inf	C_19_H_22_N_2_O_5_	B, L, F	[66]
**69**	*N*-Isopentyl-11,12-*seco*-5,6-dihydroplicane	Inf	C_23_H_30_N_2_O_5_	B, L, F	[66]
**70**	Zephycarinatine H	CNS	C_23_H_32_N_2_O_4_	B, L, F	[69]
**CRIPOWELLIN**					
**71**	4,8-Dimethoxy-cripowellin C	Inf, Mic, Oxi, Tum	C_26_H_35_NO_11_	B	[84]
**72**	4,8-Dimethoxy-cripowellin D	Inf, Mic, Oxi, Tum	C_26_H_37_NO_10_	B	[84]
**73**	9-Methoxy-cripowellin B	Inf, Mic, Oxi, Tum	C_26_H_35_NO_12_	B	[84]
**74**	4-Methoxy-8-hydroxy-cripowellin B	Inf, Mic, Oxi, Tum	C_25_H_35_NO_11_	B	[84]
**75**	Cripowellin C	Tum	C_25_H_31_NO_11_	L	[85]
**76**	Cripowellin D	Tum	C_25_H_33_NO_10_	L	[85]
**MESEMBRINE**					
**77**	Sarniensinol	Ins, Lar	C_18_H_23_NO_4_	B	[78]
**78**	Sarniensine	Ins, Lar	C_19_H_25_NO_4_	B	[86]
**OTHERS**					
**79**	(+)-*N*-Methoxylcarbonyl-2-demethyl-isocorydione	Inf, Tum	C_20_H_17_NO_7_	B	[67]
**80**	Lycoranine D	Tum	C_15_H_15_NO_3_	B	[64]
**81**	Zephycandidine A	Tum	C_16_H_10_N_2_O_2_	B, L, F	[87]
**82**	Hymenolitatine	Tum	C_17_H_15_NO_4_	B	[88]
**83**	Zephycandidine I	CNS	C_18_H_25_NO_4_	B, L, F	[30]
**84**	Zephycandidine II	CNS	C_15_H_19_NO_2_	B, L, F	[30]
**85**	Zephycandidine III	CNS	C_17_H_19_NO_4_	B, L, F	[30]
**86**	Narcipavline	CNS	C_33_H_34_N_2_O_5_	B	[29]
**87**	Narcikachnine	nm	C_33_H_36_N_2_O_5_	B	[29]
**88**	Narcimatuline	CNS	C_33_H_34_N_2_O_5_	B	[89]
**89**	Zephycarinatine A	CNS	C_25_H_32_N_2_O_5_	B, L, F	[69]
**90**	Zephycarinatine B	CNS	C_22_H_30_N_2_O_4_	B, L, F	[69]
**91**	Zephycarinatine G	CNS	C_23_H_32_N_2_O_3_	B, L, F	[69]

Abbreviation for biological activities are CNS: central nervous system, hCAII: human carbonic isozyme II, Inf: anti-inflammatory, Ins: insecticidal, Lar: larvicidal, Mic: antimicrobial, Oxi: antioxidant, Par: antiparasitic, Tum: antitumoral, and nm: not measured. Abbreviation for organs are L: leaves, B: bulbs, F: flowers, and R: rhizomes.
